# Autonomic nervous system involvement in pulmonary arterial hypertension

**DOI:** 10.1186/s12931-017-0679-6

**Published:** 2017-12-04

**Authors:** Mylène Vaillancourt, Pamela Chia, Shervin Sarji, Jason Nguyen, Nir Hoftman, Gregoire Ruffenach, Mansoureh Eghbali, Aman Mahajan, Soban Umar

**Affiliations:** Department of Anesthesiology and Perioperative Medicine, Division of Molecular Medicine, David Geffen School of Medicine, University of California Los Angeles (UCLA), Los Angeles, CA BH 520A CHS USA

**Keywords:** Pulmonary arterial hypertension, Autonomic nervous system, Right ventricle, Sympathetic nervous system, Renin angiotensin aldosterone system

## Abstract

Pulmonary arterial hypertension (PAH) is a chronic pulmonary vascular disease characterized by increased pulmonary vascular resistance (PVR) leading to right ventricular (RV) failure. Autonomic nervous system involvement in the pathogenesis of PAH has been demonstrated several years ago, however the extent of this involvement is not fully understood. PAH is associated with increased sympathetic nervous system (SNS) activation, decreased heart rate variability, and presence of cardiac arrhythmias. There is also evidence for increased renin-angiotensin-aldosterone system (RAAS) activation in PAH patients associated with clinical worsening. Reduction of neurohormonal activation could be an effective therapeutic strategy for PAH. Although therapies targeting adrenergic receptors or RAAS signaling pathways have been shown to reverse cardiac remodeling and improve outcomes in experimental pulmonary hypertension (PH)-models, the effectiveness and safety of such treatments in clinical settings have been uncertain. Recently, novel direct methods such as cervical ganglion block, pulmonary artery denervation (PADN), and renal denervation have been employed to attenuate SNS activation in PAH. In this review, we intend to summarize the multiple aspects of autonomic nervous system involvement in PAH and overview the different pharmacological and invasive strategies used to target autonomic nervous system for the treatment of PAH.

## Overview of the autonomic regulation of heart and lungs

### The autonomic nervous system

**Fig. 1:**
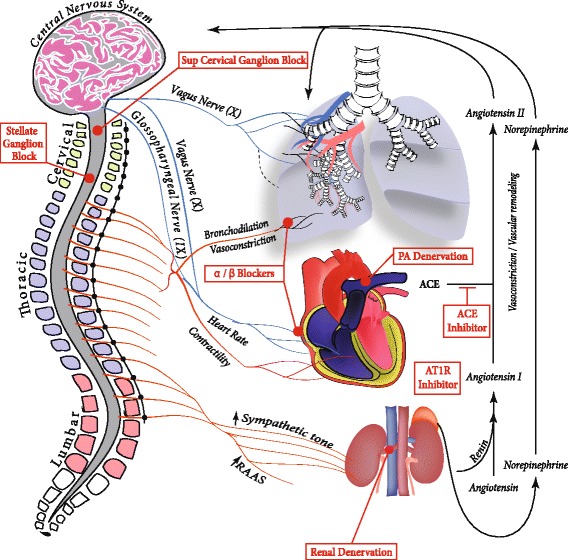
Schematic diagram summarizing autonomic nervous system involvement in PAH and various therapeutic strategies targetting the activation of SNS and RAAS in PAH. PAH is associated with increased sympathetic nervous system (SNS) and renin-angiotensin-aldosterone-system (RAAS) activation. The central nervous system provides autonomic output to the lungs and heart mainly through cranial nerves IX and X. The parasympathetic nervous system (shown in blue) originates from cranial nerves III, VII, IX, and X and the sacral nerves S2-S4. The SNS (shown in red) originates from the thoracolumbar region of the spinal cord and modulates; i) vascular and airway reactivity in the lungs, ii) heart rate and contractility in the heart and iii) RAAS activation in the kidneys and adrenal glands. Consequently, RAAS activation generates vasoactive compounds that result in pulmonary vasoconstriction and vascular remodeling, hallmarks of PAH. These vasoactive compounds may result in a feedback loop to the nervous system. Various pharmacological (α/β blockers, ACE inhibitors, AT1R inhibitors), surgical (pulmonary artery denervation (PADN), renal artery denervation) and experimental (Superior cervical and stellate ganglion block (SGB) approaches for modulating autonomic nervous system and RAAS are also shown boxed in red

The autonomic nervous system is composed of sympathetic and parasympathetic divisions and is often divided by neural and endocrine regulatory components. The sympathetic nervous system (SNS) originates from the thoracolumbar region of the spinal cord (Fig. 1). Short preganglionic fibers from the T1-L2 segments synapse on paravertebral or prevertebral ganglia, enabling long postganglionic fibers to innervate target organs such as the heart and lungs. On the other hand, the parasympathetic nervous system originates from cranial nerves III, VII, IX, and X and the sacral nerves S2-S4. In general, parasympathetics cause vasodilation of blood vessels including the pulmonary vasculature, and sympathetics cause vasoconstriction [1] (Table 1).

**Table 1:** Adrenergic, cholinergic, and angiotensin receptors and the effect of their activation in heart and lungs

Receptors	Receptor Sub-types	Activators	Heart	Lung
Adrenergic	α	• Phenylephrine [79] • Epinephrine • Norepinephrine	• Increases rate of contraction (chronotropic) [80] • Selective α_1_-AR stimulation is negatively inotropic in RV trabeculae but positively inotropic in LV trabeculae [81]	• Increases pulmonary vascular resistance [7] • Decreases compliance
	β_1_	• Dobutamine [79] • Epinephrine • Norepinephrine	• Increases rate of contraction (chronotropic) [80] • Increases force of contraction (inotropic) • Increases excitability (predisposes to arrhythmia) • Increases AV nodal conduction velocity • LV and RV are equally inotropically responsive to selective β_1_-adrenergic receptor agonism [81]	• Decreases pulmonary vascular resistance [82]
	β_2_	• Procaterol [83] • Terbutaline • Salbutamol	• Increases cardiac contractility [83] • Increases inotropy • Does not predispose to arrhythmia • Increases fatty acid modulation and glucose metabolism. • Decreases myocardial apoptosis	• Dilation of bronchi and bronchioles [80] • Decreases pulmonary vascular resistance [82]
Cholinergic	Muscarinic	• Acetylcholine [84] • Pilocarpine	• Decreases chronotropy [85] • Decreases inotropy • Decreases dromotropy • Expression of M(2,4) AChR in the right ventricle is higher than in the left ventricle [86]	• Decreases pulmonary vascular resistance via M3 receptor [84]
	Nicotinic	• Acetylcholine	• Expression of α7-nAChR is higher in the left ventricle and right ventricle than the atria [86]	• Decreased pulmonary vascular resistance (α7-nAChR via NO pathway) • Promotes cell proliferation via MAPK pathway and neoangiogenesis via increased VEGF in pulmonary endothelium [87]
Angiotensin	AT1	• Angiotensin II	• Increases chronotropy [88] • Increases inotropy • Facilitates presynaptic release of noradrenaline from cardiac sympathetic nerve terminals • Causes coronary vessel vasoconstriction • Stimulates aldosterone release • Causes myocyte hypertrophy, non-myocyte proliferation, and interstitial fibrosis	• Increases pulmonary vascular resistance [62] • Promotes proliferation, inflammation, and fibrosis in the vasculature and lung parenchyma
	AT2	• Angiotensin II	• Counteracts effects of AT1 receptor in cardiac hypertrophy and remodeling – cardioprotective [88] • Involved in cell growth, proliferation, differentiation, migration, and apoptosis • Not clear if AT2 agonism has acute effects on vascular tone	• Decreases pulmonary vascular resistance [62] • Decreased inflammation • Diminished fibrosis • Promotes cell differentiation and apoptosis • Reduces cell proliferation

### Autonomic innervation of the pulmonary vasculature

The pulmonary vasculature is innervated by sympathetic, parasympathetic, and sensory nerve fibers. Increased vascular resistance is mediated by α-adrenoreceptors upon sympathetic nerve stimulation [2]. Noradrenergic fibers are activated by baroreceptors in the pulmonary artery [3] and proximal airway segments [4]. Chemoreceptors respond to decreased arterial PO2 levels to increase sympathetic nerve stimulation by the sympathetic chain neurons [2, 5]. Parasympathetic activation via vagal stimulation results in cholinergic-mediated relaxation of pulmonary arteries [6]. Many other factors (i.e. non-adrenergic and non-cholinergic mediators, peptides, trophic factors, differential release of transmitters by high or low frequencies) are implicated in sympathetic and parasympathetic regulation of lung vasculature, though their functions have not entirely been elucidated [7] (Table 1).

### Autonomic innervation of the heart

The heart is also innervated by both parasympathetic and sympathetic fibers (Fig. 1). The parasympathetic fibers are responsible for decreasing chronotropy, dromotropy, and inotropy via cholinergic action on cardiac M2 receptors. The SNS acts on β1 adrenergic receptors to increase chronotropy, dromotropy, and inotropy of the heart [8]. Interestingly, β-adrenergic stimulation has been shown to have a significantly greater positive inotropic effect on left ventricular (LV) contractility than on right ventricular (RV) contractility [9]. On the contrary, adrenergic stimulation of alpha 1 receptors result in increased inotropy in the LV but decreased inotropy in the RV [10] (Table 1).

Patients with PAH often have normal systemic blood pressures and lung volumes. However, they may suffer from hypoxia, hypercarbia, acidosis, and, in later stages, RV hypertrophy and failure. Neural pathways controlling the heart and lungs are described in detail within current scientific literature [11].

### Autonomic nervous system and RAAS involvement in PAH

PAH is a clinical syndrome characterized by pathologic pulmonary 1) vasoconstriction, 2) vascular remodelling, and 3) thrombosis. Progressive sequelae include increased pulmonary vascular resistance (PVR), RV hypertrophy and dysfunction, and ultimately death. The extent of involvement of the autonomic nervous system in the pathogenesis of PAH is not fully understood. It is postulated that the patients with PAH often have a low cardiac output and may compensate for that by upregulation of neurohormonal systems such as SNS and renin-angiotensin-aldosterone system (RAAS) [12] (Fig. 1). Inflammatory and RAAS molecules are specifically upregulated in PAH and are implicated in the development of the disease through their effects on the brain [13].

There is an increasing body of evidence linking autonomic nervous system involvement in the pathogenesis of PAH. Here we review the available literature on SNS activation, heart rate variability, baroreflex sensitivity and arrhythmias, and RAAS dysregulation in PAH. We also summarize the therapeutic strategies for modulating the autonomic nervous system and RAAS in PAH.

## Sympathetic nervous system activation in PAH

Over the last 30 years, accumulating evidence has supported the involvement of the autonomic nervous system in PAH, strengthening the hypothesis for the role of SNS activation in PAH development. Microneurography was used to compare the muscle sympathetic nerve activity (MSNA) between patients with PAH and healthy controls and showed increased sympathetic nerve traffic in PAH patients [14]. MSNA was also directly correlated with heart rate, presence of pericardial effusion, oxygen saturation, New-York Heart Association class, 6-min walk distance (6-MWD), and pulmonary arterial acceleration time and was associated with clinical deterioration [14, 15]. Administration of hyperoxia decreased MSNA frequency and burst amplitude, suggesting that peripheral chemoreceptors contribute in part to increased MSNA [14].

Increased SNS activity in PAH was also assessed by neurohormonal activation, despite conflicting results between studies. In a small clinical study comprising 32 patients with PAH, plasma norepinephrine concentration was strongly correlated to PVR and was associated with poor estimated 5-year survival [16]. Some other studies have shown an increase in plasma norepinephrine concentration in PAH patients compared to healthy controls, although these norepinephrine levels did not exceed normal values and were not correlated with any hemodynamic parameter [17, 18]. To determine the involvement of catecholamine pathway in PAH, many groups have directed their research towards catecholamine receptors.

Bristow and colleagues [19] were the first to describe local changes in β-adrenergic receptors in the failing RV myocardium of PAH patients. Since then, the impact of β-adrenergic receptor signaling in PAH development has been extensively studied using different α/β-adrenergic receptor agonists/antagonists. Ishikawa and colleagues [20] showed that administration of arotinolol, a pure α/β-adrenergic receptor antagonist, prevents monocrotaline (MCT)-induced PH development by keeping cardiopulmonary pressures below pathological threshold and decreasing RV/body weight ratio in treated rats. Treatment with the nonselective adrenergic receptor antagonist carvedilol was also reported to reverse established RV failure in two different rat models of PH (Sugen/hypoxia and MCT-induced PH) and improved survival in MCT rats [21, 22]. This improvement in RV function was associated with reduced RV hypertrophy, dilation, and fibrosis, as well as an improved capillary density of the myocardium [21]. Interestingly, carvedilol also decreased pro-fibrotic signaling and extracellular matrix remodelling in right and left ventricles of treated rats via transforming growth factor-β1, connective tissue growth factor, SMAD2/3, p38, and metalloproteinases 2 and 3 pathways. Drake and colleagues [23] performed an extensive microarray gene expression analysis on the RV of Sugen/hypoxia PH rats either treated with carvedilol for 4 weeks or untreated. Within the top canonical pathways revealed in this analysis, one was involved in cardiac hypertrophy by protein synthesis and included regulation of eukaryotic initiation factor 4 and 5 and p70S6k signaling, all of which were downregulated with carvedilol. Ceramide signaling and glucocorticoid receptor signaling pathways, considered injurious for the heart, were also two of the top five canonical pathways and were downregulated in carvedilol-treated rats. Other canonical pathways included the peroxisome proliferator-activator receptor signaling, peroxisome proliferator-activator receptor/retinoid X receptor-activation, and nuclear respiratory factor 2-mediated oxidative stress response. These pathways are involved in metabolism, mitochondrial function, and oxidative stress response and are critical for adequate heart function. All these findings highlight the role of adrenergic receptors in RV failure and suggest that the use of β-blockers could be beneficial for PAH patients. In the clinical settings, carvedilol showed encouraging results by improving RV ejection fraction in chronic heart failure [24] and did not lead to any notable adverse event or deterioration [25, 26] (Table 2). In a recent 6-month double-blind, randomized, controlled trial, patients treated with carvedilol had an improved heart rate recovery after exercising compared to those who received placebo [26]. This is important since heart rate recovery after exercise is not only a predictor of increased risk of death but also of clinical worsening in PAH. RV function was also assessed by an improved glycolytic rate related to carvedilol treatment. [26] Clinical trials are still ongoing to confirm the therapeutic benefit and safety of carvedilol in PAH patients (ClinicalTrials.gov Identifier: NCT02120339 and NCT02507011).

**Table 2:** Clinical trials on neurohormonal modulation in pulmonary hypertension

Study	Treatment	Study design	Follow-up	Subjects	Patient description (n patient)	Changes in outcomes	Related side effects (n patient)
Pharmacological therapies							
Grinnan and colleagues [25]	Carvedilol	Single-arm, open-label, pilot	6 months	6	iPAH (3) HPAH (1) APAH (2)	↑RVEF ↑BNP level	Bradycardia (1) Asymptomatic hypotension (1) Mild fatigue (1)
Farha and colleagues [26]	Carvedilol	Single-center, double-blind, randomized, controlled trial	6 months	30	iPAH (9) HPAH (12) APAH (5) PH due to lung disease and/or hypoxemia (2) Chronic thromboembolic PH (2)	↓HR ↓ RVSP at 3 month follow-up ↑ RV fractional area change at 3 month follow-up ↑RV glycolytic rate ↑β-adrenergic receptor density	Fatigue (1) Dyspnea (2) Leg swelling (3) Site infection/acute bronchitis (2) Chest pain (1) Blurry vision (1) Cholecystitis (1) Bloating (1) Nausea/vomiting (1) Dizziness (1)
van Campen and colleagues [29]	Bisoprolol	Prospective, randomised, placebo-controlled, crossover	1 years	18	iPAH (18)	↓HR ↑PAWP ↓Cardiac index ↓6-MWD	Worsening of fluid retention (1) Hypotension (1) Tiredness (1) Feelings of depression (1)
Bandyopadhyay and colleagues [31]	Atenolol Bisoprolol Carvedilol Metaprolol Nebivelol Propranolol Sotalol	Retrospective cohort	5 years	568	iPAH (260) APAH (308)	None reported	Therapy discontinued (60): Hypotension (18) Shortness of breath (14) Volume overlaod (12) Fatigue (7) Bradycardia/Syncope (4) Intolerance (3) Other (2)
Moretti and colleagues [32]	Atenolol Bisoprolol Metaprolol Nadolol Propranolol	Prospective cohort	2 years	94	iPAH (14) APAH (27) Post pulmonary embolism (13) Out of proportion (8) Mixed (5) Unknown (27)	↑TAPSE ↓RV diameter ↓HR ↓Systolic blood pressure	None reported
So and colleagues [33]	Acebutolol Atenolol Bisoprolol Metaprolol Nadolol Propranolol	Prospective cohort	2 years	94	iPAH (53) Drug/toxin-iduced PAH (2) APAH (39)	↑Cardiac index	None reported
Thenappan and colleagues[34]	Atenolol Carvedilol Labetalol Metaprolol Nadolol Propranolol	Retrospective cohort study, propensity score analysis	5 years	564	iPAH (250) HPAH (17) Drug/Toxin-induced (21) APAH (273) Other (3)	None reported	None reported
Bozbas and colleagues [60]	Losartan Nifedipine	Prospective, randomized	2 months	63	PH due to left heart disease (40) PH due to lung disease and/or hypoxemia (23)	↓mean PAP ↑RVEF ↑6-MWD ↓VE ↓VE/VCO_2_ ↑PETCO_2_ ↑CPET test duration	None reported
Maron and colleagues [69]	Spironolactone ±Ambrisentan	Retrospective analysis of randomized, placebo-controlled trials ARIES-1 and 2	2 months	199	Primary PAH (126) Nonprimary PAH (73)	↑6-MWD ↓BNP level ↓WHO class	Pulmonary hypertension (21) Edema or prevention of edema (11) RV failure (7) Electrolyte imbalance (1)
Invasive strategies							
Chen and colleague [74]	PADN	Prospective cohort	3 months	21	iPAH (21)	↓mean PAP ↓PVR ↓RVSP ↓TPG ↓Pericardial effusion ↑PA compliance ↑6-MWD ↓WHO class ↓NT-proBNP level ↓Rehospitalization	Chest pain (10)
Chen and colleagues [75]	PADN	Prospective cohort, single-arm	1 year	66	iPAH (20) APAH (19) PH due to left heart disease (18) Chronic thromboembolic PH (9)	↓mean PAP ↓PVR ↓RVSP ↑Cardiac output ↓Right atrial diameter ↓RV diameter ↓pericardial effusion ↑6-MWD ↓NT-proBNP level	Chest pain (47) Sinus bradycardia (1) Intolerance to dyspnea (3)

*6-MWD* 6-min walk distance, *APAH* associated pulmonary arterial hypertension, *BNP* brain natriuretic peptide, *CPET* cardiopulmonary exercise testing, *HPAH* heritable pulmonary arterial hypertension, *HR* heart rate, *iPAH* idiopathic pulmonary arterial hypertension, *NT-proBNP* N-terminal pro-brain natriuretic peptide, *PA* pulmonary artery, *PAH* pulmonary arterial hypertension, *PAP* pulmonary arterial pressure, *PAWP* pulmonary artery wedge pressure, *PETCO*_*2*_ end-tidal carbon dioxide tension, *PH* pulmonary hypertension, *PVR* pulmonary vascular resistance, *RV* right ventricle, *RVEF* right ventricular ejection fraction, *RVSP* right ventricular systolic pressure, *TAPSE* tricuspid annular plane systolic excursion, *TPG* transpulmonary pressure gradient, *VCO*_*2*_ volume of carbon dioxide production, *VE* pulmonary ventilation, *WHO class* World Health Organization Class

De Man and colleagues [27] investigated the use of bisoprolol, a cardioselective β-adrenergic receptor blocker, in the progession of RV failure in MCT-induced PH model. They showed that RV failure progression was significantly delayed in bisoprolol treated rats with an improvement in RV contractility and filling, a partially recovered cardiac output, and decreased RV interstitial fibrosis and myocardial inflammation. Bisoprolol restored β-adrenergic receptor signaling assessed by increased phosphorylation of its downstream targets, myosin binding protein C and troponin I. In contrast, decreased phosphorylation of these proteins in PAH cardiomyocytes leads to an increase in sarcomere Ca^2+^ sensitivity, thus impairing RV relaxation and contributing to RV stiffness [28]. However, these beneficial effects were not confirmed in an explorative study involving 18 PAH patients (Table 2) [29]. In this randomized, placebo-controlled, crossover study, bisoprolol did not improve patients’ conditions. Despite a trend to increase RV ejection fraction, patients had a significant decrease in cardiac index and a near significant drop in 6-MWD, demonstrating no real benefit of bisoprolol in PAH (Table 2).

Finally, Perros and colleagues [30] compared the effects of nebivolol, a third generation β-adrenergic receptor blocker, to the first generation β1-adrenergic receptor blocker metoprolol in MCT-induced PH rats. Nebivolol is a β1-adrenergic receptor antagonist and β2,3-adrenergic receptor agonist and has vasodilator properties in addition to its adrenergic-modulating characteristics. Daily administration of nebivolol for one week in established PH improved cardiopulmonary hemodynamics and partially reversed RV hypertrophy and pulmonary vascular remodeling with a greater effect than metropolol. In vitro nebivolol, but not metropolol, significantly decreased human pulmonary endothelial cell proliferation as well as the production of pro-inflammatory cytokines such as interleukin-6 and monocyte chemoattractant protein-1, epidermal and fibroblast growth factors, and the vasoconstrictor endothelin-1. Furthermore, human smooth muscle cell proliferation was decreased when cells were cultured in the endothelial cell + nebivolol-conditioned media [30]. A clinical trial is currently recruiting patients to assess the therapeutic potential and safety of nebivolol in clinical managment of the PAH patients (ClinicalTrials.gov Identifier: NCT02053246).

Despite encouraging results of adrenoreceptor blockade in experimental PH, the use of β-blockers in clinical PAH is still largely debated due to the poor beneficial results as well as safety concerns revealed in clinical trials [31–36] (Table 2). Although β-blockers partially reverse RV structural and molecular remodeling [23, 24] and seem beneficial and well-tolerated at low or escalating doses [25, 26, 29, 31–34], their negative inotropic and chronotropic effects may actually impair RV function in severe heart failure. Thus, the choice based on the specificity of the β-blocker used to target one or the other β-receptor sub-type may be of importance. The divergent results found in clinical studies may also be the consequence of several limiting factors, such as study design, cohort size, diversity in pulmonary hypertension etiology within cohorts, and variability in pre-existing comorbidities and treatments taken by patients.

## Heart rate variability and baroreflex sensitivity in PAH

Heart failure is associated with abnormalities in autonomic nervous system control, including decreased heart rate variability and blunted baroreflex sensitivity [37], which were predictors of cardiovascular mortality among post-myocardial infarction patients [38, 39]. Considering the evidence of an altered autonomic control in PAH, these two parameters were investigated in different PAH cohorts. In a small cohort of 9 PAH patients, heart rate variability was assessed by a time-domain method and was found to be significantly decreased compared to the control group (*n* = 20) [40]. In a larger cohort, Wensel and colleagues [41] used a frequency-domain method to show a reduction of total power of heart rate variability in PAH patients (*n* = 42) compared to healthy subjects (*n* = 41) but without any significant difference between low/high frequency power ratio. Baroreflex sensitivity was also reduced in PAH patients, determined by the controlled breath method and the α-index of low frequency. Both heart rate variability and baroreflex sensitivity parameters were correlated with decreased peak oxygen uptake during exercise, demonstrating the impact of cardiac autonomous modulation in exercise capacity [41]. In a cohort of 47 children with severe PAH, heart rate variability was correlated with 6-MWD and was predictive of transplantation and/or mortality [42]. On bivariate analysis, standard deviation of mean values for the beat-to-beat interval over 5 min predicted outcome independently of functional status, syncope, RV function, and haemodynamic parameters [42]. More recently, Yi and colleagues [43] also confirmed that time-domain heart rate variability parameters, as well as frequency-domain indices, were significantly decreased in PAH patients (*n* = 26) compared to the controls (*n* = 51). Furthermore, heart rate variability parameters correlated to mean pulmonary arterial pressure (PAP) in patients [44, 45]. Further studies in larger patient cohorts are needed to confirm if the heart rate variability and baroreflex sensitivity changes seen in PAH are indeed associated with a decrease in the vagal tone and whether they carry prognostic significance.

## Cardiac arrhythmias in PAH

In experimental PH, our group and others have described spontaneous ventricular fibrillation events and have extensively investigated their mechanisms [46–48]. Benoist and colleagues [46] showed in isolated hearts that the monophasic action potential duration at 25, 50 and 90% repolarization was significantly prolonged in hearts from MCT-induced PH rats compared to controls for both RV and LV. Futhermore, RV monophasic action potential duration at 90% repolarization strongly correlated with cardiac hypertrophy. These changes in the action potential duration were associated with decreased K^+^ and L-type Ca^2+^ channel expressions as well as an increase in T-type Ca^2+^ channels in the RV of MCT rats [46]. In a subsequent study, the same group monitored MCT rats using electrocardiograms and showed an increased QT interval in RV failure from day 15 post-MCT injection consistent with the prolonged action potential duration [47]. Alternans of both T-wave amplitude and QT interval were also observed in animals with heart failure. In isolated hearts, alternans occurred in four out of six failing hearts and were always discordant. Action potential duration alternans have been associated with dysfunctional Ca^2+^ homeostasis, resulting in increased intracellular Ca^2+^ concentration and contractile alternans. Ca^2+^ transient alternans were confirmed in isolated myocytes from failing RVs and were shown to be provoked by dysregulation of sarcoplasmic Ca^2+^ uptake, load, and release. Consistent with this, failing RVs of MCT rats showed a reduced SERCA2a activity, an increased sarcoplasmic reticulum Ca^2+^ release fraction, and an increased Ca^2+^ spark leak. [47].

In MCT rats, our group [48] showed that the onset of ventricular fibrillation was preceded by early afterdepolarizations, triggering an activity which caused a non-sustained ventricular tachycardia which then degenerated to ventricular fibrillation. PH-induced RV failure was associated with RV epicardial and endocardial fibrosis, which is known to promote early afterdepolarizations by cardiomyocytes and cause ventricular fibrillation. In addition to fibrosis, we proposed that a selective downregulation of Kv1.5, KCNE2, and SERCA2a in failing RV may lead to reduction of RV myocyte repolarization reserve, which could also facilitate the formation of early afterdepolarizations [48]. These episodes of ventricular fibrillation were detected between days 23 and 32 after MCT injection, corresponding to a period of expected sudden death in these animals. We also showed a 10-day window between the drop of RV ejection fraction (from ~72% at day 14 to ~38% at day 21) and the beginning of this sudden death period, suggesting that mortality during this critical period may be caused by early afterdepolarizations-mediated triggered activity in the RV [48].

In clinical PAH, cardiac arrhythmias are common in patients and are associated with worsening prognosis. Cardiac arrhythmias include supraventricular arrhythmias (SVA), which are the most common with a reported incidence between 11 and 30% [49–53], and the ventricular arrhythmias, which were reported to be relatively rare in PAH [40]. The most common SVAs seen in PAH patients are atrial fibrillation, flutter, and tachycardia [49, 51, 53]. In a 6-year retrospective single-center analysis including 231 PAH patients, 31 SVA episodes were detected in 27 patients (cumulative incidence 11.7%, annual risk 2.8% per patient) [49]. In most episodes (*n* = 26), onset of supraventricular tachycardia resulted in clinical deterioration and/or RV failure. Furthermore, mortality was significantly higher in patients with persistent atrial fibrillation compared with the patients in whom sinus rhythm was restored. In a 5-year prospective study including 239 patients, at least one episode of atrial flutter or atrial fibrillation was detected in 20% of the patients [50]. Compared to patients who did not develop SVA, patients who developed atrial flutter and fibrillation had significantly higher baseline values for right atrial pressure, mean PAP, PVR, lower baseline values for cardiac output, and mixed venous oxygen saturation. Futhermore, the estimated 1, 2, 3, and 5 year survival rates after diagnosis of PH in patients with permanent atrial fibrillation were 80, 66, 22, and 22%, respectively [50]. Presence of SVA in PAH patients is also associated with deterioration of the RV function, assessed by an increase in B-type natriuretic peptide, atrial and ventricular diameter, mean atrial pressure, and PVR, as well as a reduction of the cardiac index [51]. The estimated survival rate is lower in patients who develope SVA, and is significantly worse in patients with permanent SVA compared to transient or without SVA [51, 52]. A good understanding of cardiac arrhythmias and other autonomic nervous system dysregulations is important not only because they are determinants of the outcome for the patients, but they also determine the clinical management of PAH in these patients [49, 53].

## Renin-angiotensin-aldosterone-system dysregulation in PAH

**Fig. 2:**
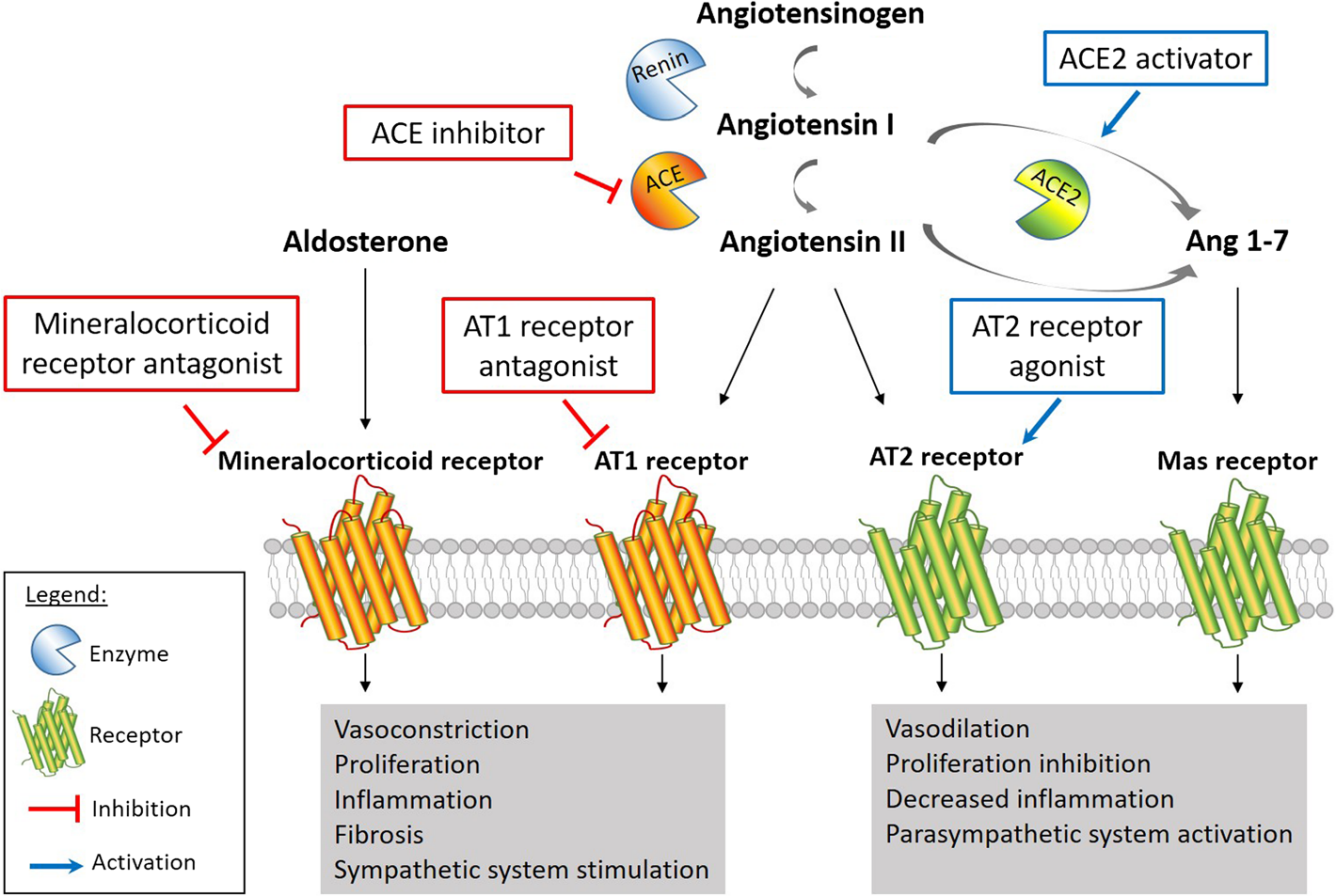
Renin-angiontensin-aldosterone system (RAAS) activation in PAH. Renin cleaves angiotensinogen to angiotensin I, which is further processed by the angiotensin-converting enzyme (ACE) to the biologically active peptide angiotensin II and binds to angiotensin receptors AT1 and AT2. Angiotensin I and II may also undergoe further processing by ACE2 to yield angiotensin (1–7), which activates the Mas receptor. On the other hand, aldosterone activates the mineralocorticoid receptors. Both AT1 and mineralocorticoid receptor activation lead to pathological signaling in PAH, and targeting these pathways using receptor antagonists or ACE inhibitors improves PAH. On the other hand, AT2 and Mas signaling are protective, and promoting these signaling cascades using AT2 agonists or ACE2 activators improves autonomic nervous system imbalance seen in PAH

Chronic activation of the RAAS in PAH patients is well described. As extensively reviewed by Maron and Leopold, RAAS activation promotes pulmonary vasoconstriction, cell proliferation, migration, extracellular matrix remodeling and fibrosis resulting in pulmonary vascular remodeling in experimental PH (Fig. 2) [54].

### Renin, angiotensin I, and angiotensin II

In PAH patients, circulating renin activity as well as plasma angiotensin I and II are significantly up-regulated and associated with disease worsening, suggesting a role for systemic RAAS activation in PAH progression [12, 55]. Futhermore, plasma renin and angiotensin II were strongly associated with an increased risk of death or lung transplant, making them potentially reliable biomarkers for clinical prognosis [12]. To determine whether local RAAS activation was also involved in PAH, de Man et al. [12] investigated angiotensin II signaling pathway in human PAH lung tissues. They found a two-fold increase of angiotensin II receptor AT1, but not AT2, in the pulmonary vasculature of PAH patients along with an increase in activity of its downstream targets Src and ERK. This is an important finding given that AT1 signaling is involved in vasoconstriction, oxidative stress, inflammation, and proliferation, while AT2 signaling leads to vasodilation and is vasculoprotective (Fig. 2) [54]. Exposing pulmonary endothelial cells to angiotensin I revealed a significatly increased angiotensin II production by cells isolated from PAH patients compared to control, which was abolished with the angiotensin-converting enzyme (ACE) inhibitor enalapril [12]. This high angiotensin II production by endothelial cells induces smooth muscle cell proliferation, resulting in pulmonary vascular medial hypertrophy and obliteration. In vivo, pharmacological AT_1_ receptor antagonism using losartan significantly delayed disease progression in MCT-induced PH rats by reducing RV afterload, restoring ventricular–arterial coupling, and improving RV diastolic function. Furthermore, losartan significantly reduced pulmonary vascular remodeling in treated-rats but without any change in RV hypertrophy [12]. However, these results are inconsistent with previous studies in MCT rats using the same AT_1_ antagonist, in which researchers did not find any prophylactic effect of losartan against the development of PAH [56, 57]. Two independent groups also tested losartan in a pressure-overload right heart failure model. Borgdorff and colleagues [58] treated pulmonary artery banded (PAB)-rats with combined losartan + eplerenone treatment until RV failure criteria were met or for a maximum of 11 weeks. Combined losartan + eplerenone treatment did not prevent adverse RV remodeling or clinical RV failure. Interestingly, treated PAB rats did have a significant decrease in LV peak and aortic pressures, highlighting inherent differences between right and left ventricles and their significance in research for therapies in the context of PAH [58]. PAB model was also used to compare the preventive effect of losartan and bisoprolol on development of RV hypertrophy and dysfunction [59]. After 6 weeks of treatment with either losartan or bisoprolol, rats did not show any signs of improvement in RV hypertrophy, dysfunction, fibrosis or capillary density. Furthermore, neither losartan nor bisoprolol reversed gene expression levels of cardiac hypertrophy and dysfunction biomarkers.This study is additional evidence of how the RV differs substantially from the LV when responding to inhibition of the increased neurohormonal activation occurring in heart failure.

In the clinical setting, 33 PH patients from different etiologies were followed after 8 week- treatment of losartan (Table 2) [60]. A modest but significant decrease in mean PAP and increase in RV ejection fraction were observed in subjects taking losartan compared to their baseline. They also found an improvement in 6-MWD and in several cardiopulmonary exercise testing parameters. However, the duration of treatment was relatively brief, and the study was lacking a control group. Furthermore, since the cohort was comprised of subjects from different PH classification groups, further studies are needed to assess the real potential of losartan in PAH patients.

### ACE2/Angiotensin-(1–7)/Mas axis

Another method to counter the vasoconstrictive and proliferative ACE/angiotensin II/AT1 receptor axis in PAH was demonstrated by activating the vasoprotective ACE2/Angiotensin-(1–7)/Mas axis of the RAAS. Ferreira and colleagues [61] administered the compound XNT, a synthetic activator of ACE2, in MCT-induced PH rats during the 28 days of the protocol. XNT significantly decreased RV pressure and hypertrophy in treated rats. This improvment was abolished when XNT was co-administrered with A779, a Mas antagonist, supporting the hypothesis that beneficial effects of ACE2 activation would be mediated by an increase in Ang-(1–7) levels to shift the balance from the ACE/angiotensin II/AT1 receptor axis toward the ACE2/Angiotensin-(1–7)/Mas axis of the RAAS. MCT treatment alone caused significant increases in renin and angiotensinogen mRNA as well as in the AT1 receptor and ACE mRNA levels, all of which were reversed with XNT treatment. ACE2 activation also significantly attenuated the mRNA levels of the inflammatory mediators tumor necrosis factor-α, interleukin-1, interleukin-6, monocyte chemoattractant protein-1, as well as nuclear factor-kappa B p50 and p65 [61]. Similar results were found when MCT rats were treated with compound C21, an AT2 receptor agonist, suggesting an endogenous protective role of AT2 receptors in PH [62]. Interestingly, either AT2 receptor or Mas blockade prevented the protective effect of the C21, suggesting a connection between both receptors. The same group showed that daily oral administration of 500 mg of bioencapsulated ACE2 or angiotensin-(1–7) prevents and rescues MCT-induced PH, with a greater effect when both therapies were combined [63]. ACE2 and angiotensin-(1–7) improved RV function, and decreased pulmonary vascular wall thickness and inflammatory markers as well as autophagy assessed by the reduction of LC3B-II protein level. Li and colleagues [64] demonstrated the prevention of PH using the pharmacological compound resorcinolnaphthalein, an ACE2 activator, in a severe PH model. Briefly, they injected a single dose of MCT (40 mg/kg) 1 week after performing pneumonectomy in rats. At the time of MCT injection, they implanted osmotic minipumps containing either resorcinolnaphthalein or the vehicle for 21 days of infusion. ACE2 activation decreased mean PAP and subsequent RV hypertrophy without any effect on systemic pressure. This change in mean PAP was at least in part due to a partial restoration of acetylcholine-induced pulmonary vasorelaxation. Resorcinolnaphthalein also decreased the neointimal formation in small pulmonary arteries, from 95% in MCT + pneumonectomy group to 29% in treated rats. In accordance with the findings of Ferreira and colleagues [61], beneficial effects of ACE2 activation in PAH seem to be at least partly mediated through angiotensin-(1–7)/Mas axis, since improvements of mean PAP, RV hypertrophy, pulmonary vasorelaxation, and neointimal formation were all abolished by the Mas antagonist A779 [64]. Finally, the role of ACE2/angiotensin (1–7)/mas axis in autonomic nervous system modulation was demonstrated using diminazene aceturate, a putative angiotensin 1–7 converting enzyme activator [65]. Diminazene treatment resulted in significant improvement in power spectrum parameters, such as normalized high and low frequency components in treated rats compared to MCT alone, thus reversing the imbalance in the autonomic nervous system modulation seen in PH. Clinical trials are now in progress to assess mechanism, safety, and efficacy of ACE-2 treatment for PAH patients (ClinicalTrials.gov Identifier: NCT01884051 and NCT03177603).

### Aldosterone

Another key player of RAAS is the steroid hormone aldosterone. Maron and colleagues [66] found an increase of aldosterone levels in plasma and lung tissues of MCT- and Sugen/hypoxia-induced PH animals. Elevated aldosterone levels were mediated by endothelin-1 via peroxisome proliferator-activated receptor gamma coactivator-1α/steroidogenesis factor-1, and increased oxidative stress in pulmonary artery endothelial cells, leading to an inhibition of nitric oxide production. Both in preventive and rescue protocols, spironolactone, a mineralocorticoid receptor antagonist, improved RV hypertrophy, PAP, PVR, and pulmonary artery remodeling along with decreased reactive oxygen species generation and restoration of nitric oxide production [66]. The therapeutic effect of spironolactone was further confirmed in a chronic hypoxia-induced PH mouse model [67]. Furthermore, the latest study demonstrated that mineralocorticoid receptor, once activated by aldosterone, induces transcriptional activity and proliferation in pulmonary arterial smooth muscle cells, which was prevented by spironolactone [67].

In a clinical study, Maron and colleagues [68] confirmed hyperaldosteronism in a small cohort of PAH patients.. Among controls (*n* = 5) and treatment-naïve PAH patients (*n* = 6), aldosterone levels also positively correlated with PVR and transpulmonary gradient and inversely correlated with cardiac output. Maron and colleagues [69] also analyzed the data from patients in whom spironolactone use was reported during ARIES-1 and -2 studies, which were randomized, double-blind, placebo-controlled trials assessing the effect of the endothelin receptor antagonist ambrisentan for 12 weeks on clinical outcomes in PAH (Table 2). Compared to patients treated with ambrisentan alone (*n* = 57), patients in whom spironolactone therapy was reported during the trial (*n* = 10) had a trend toward further improvement in 6-MWD, a 1.7 fold increase in B-type natriuretic peptide plasma levels, as well as improvement in World Health Organization functional classification. However, a recent and larger study showed no association between B-type natriuretic peptide levels, 6-MWD, Borg dyspnea score, RV systolic pressure, cardiac output, or cardiac index [70]. Aldosterone levels were also not associated with mortality. This discrepancy highlights the need for further investigations on larger cohorts to define the usefulness of aldosterone antagonism in PAH. Clinical trials are actually ongoing to define the effect, safety and tolerability of the use of aldosterone antagonists in PAH (ClinicalTrials.gov Identifier: NCT01468571, NCT01712620, NCT02253394).

## Invasive strategies for modulating the autonomic nervous system in PAH

### Sympathetic ganglion block for the attenuation of experimental PH

The activation of the SNS in PAH is well recognized, and, as seen previously, different methodologies have been considered to decrease this activation and improve PAH. Na and colleagues [71] investigated the therapeutic potential of a sympathetic ganglion block (SGB) in experimental PH (Fig. 1). Briefly, two weeks after MCT injection in rats, they administered daily injections of either saline or ropivacaine, a local anesthetic, into the left superior cervical ganglion for 14 days. Compared to MCT rats treated with saline, SGB significantly decreased RV pressures, RV hypertrophy, and pulmonary arterial wall thickness. This improvement was associated with a switch in endothelial nitric oxide synthase and arginase activity in rats injected with ropivacaine compared to those injected with saline, resulting in an increase in lung cGMP and plasma nitrite levels [71]. Finally, SGB decreased pulmonary oxidative stress, assessed by a restoration of superoxide dismutase activity and decreased malondialdehyde and nitrotyrosine levels in the lung tissues. These findings support SGB as a potential novel therapeutic approach to treat PAH.

### Pulmonary artery denervation for the treatment of PAH

Baroreceptors and sympathetic nerve fibers are localized in or near the bifurcation area of the main pulmonary artery. Chen and colleagues [72] first tested pulmonary artery denervation (PADN) in baloon-occlusion-induced PAH by occluding the left pulmonary interlobar artery in 10 Mongolian dogs. Five minutes after the occlusion, the mean absolute hemodynamic changes reached their peak with mean PAP Δ16.6 mmHg, RVSP Δ14.1 mmHg, and PVR Δ1.144 dynes/s/cm^5^ compared to baseline. These changes at five minutes were completely abolished with the PADN treatment compared to baseline. Recently, the nerve distribution around the pulmonary artery has been investigated in a swine model to determine the effect of radiofrequency PADN on acute PH induced by vasoconstriction using thromboxane A_2_ agonist [73]. Mean PAP was significantly decreased following thromboxane A_2_ agonist injection in swine treated with PADN (*n* = 4) compared to the sham group (n = 4). This change correlated with the number of histological denervation lesions in pulmonary arteries. Furthermore, they demonstrated that the depth of histological changes induced by radiofrequency energy delivery varied with anatomic location and wall thickness, indicating that the location is critical to successful PADN.

Chen and colleagues [74] tested for the first time the safety and efficacy of PADN intervention in patients with PAH (PADN-1 study). At 3 months follow-up, patients who underwent PADN procedure (*n* = 13) showed significant reduction of mean PAP and PVR and significant improvement of 6-MWD, World Health Organization class, and N-terminal brain natriuretic peptide level compared to control group (*n* = 8). A few years later, a phase II of this study was performed in a cohort of 66 patients with mixed PH ethiologies who all underwent PADN treatment [75]. Compared to baseline, the 6 months follow-up confirmed an improvement of parameters cited above. None of the parameters changed between the 6 months and 1 year follow-up, confirming the maintenance of benefical effects (Table 2). These clinical studies are the demonstration of a promising new strategy for treatment of PAH. However, these results should be interpreted carefully since either there was no control group, or whenever there was one, that group did not undergo the catheter insertion (sham) procedure. Furthermore, PADN procedure should be tested in combination with current pharmacological therapies used in PAH. In summary, further clinical trials testing PADN in addition to current therapies, including a control (sham) group, are needed to assess the effectiveness and safety of PADN strategy in the treatment of PAH. Clinical trials are ongoing to assess the efficacy of this technique in PAH (ClinicalTrials.gov Identifier: NCT02516722, NCT02220335 and NCT02525926).

### Catheter-based renal denervation as a treatment of PAH

Catheter based renal denervation is an intervention that reduces activation of the SNS and the RAAS (Fig. 1) by destroying sympathetic nerve fibers of the renal periarterial nerve plexus via small bursts of radiofrequency energy along the lengh of the nerve. Qingyan and colleagues [76] reported the first preclinical results of this strategy in a proof-of-concept study that evaluated the efficacy of catheter renal denervation as a treatment for PAH in a canine MCT experimental model. Renal denervation improved cardiopulmonary hemodynamics, attenuated pulmonary vascular remodeling, and decreased myocardial fibrosis in experimental PH. At the molecular level, renal denervation reduced angiotensin II type-1, but not type 2, receptor expression in the pulmonary arterial tissue [76]. However, these beneficial effects should be analyzed cautiously, since this study lack of control-stimulation (sham) group. This is particularly important since a large, prospective, single-blind, randomized, sham-controlled trial reported a significant decrease of the systolic blood pressure in the sham group (*n* = 171) as well as in systemic hypertensive patients (*n* = 364) [77]. Very recently, a group responded to this important issue by performing renal denervation 24 h and 2 weeks after MCT injection in rats, with control groups undergoing a sham renal denervation surgery [78]. After a 35 days follow-up, they confirmed the beneficial effects of this procedure on the lung and cardiac histopathology. Interestingly, the sooner the surgery was performed during the development of the disease, the better were the results. These exciting results support a potential benefit of catheter-based renal denervation for the treatment of PAH patients.

## Conclusions

Although PAH is generally associated with increased sympathetic nervous system activation, the precise role of autonomic nervous system involvement in the pathogenesis of PAH is still not fully understood. Pharmacologic adrenergic blockade, reduction of neurohormonal activation, and novel direct methods to attenuate sympathetic nervous system activation such as renal denervation and pulmonary artery denervation may be beneficial in PAH. Further studies are needed to determine the extent of involvement of the autonomic nervous system in PAH and to assess the effectiveness and safety of targeting the autonomic nervous system and RAAS for the treatment of PAH patients.

## Abbreviations

6-MWD: 6-Minutes walk distance
ACE: Angiotensin-converting enzyme
LV: Left ventricle
MCT: Monocrotaline
MSNA: Muscle sympathetic nerve activity
PAB: Pulmonary artery banding
PADN: Pulmonary artery denervation
PAH: Pulmonary arterial hypertension
PAP: Pulmonary arterial pressure
PH: Pulmonary hypertension
PVR: Pulmonary vascular resistance
RAAS: Renin-angiotensin-aldosterone system
RVs: Right ventricle
SGB: Sympathetic ganglion block
SNS: Sympathetic nervous system
SVA: Supraventricular arrythmias
